# Tubercular Phthisis; the Result of Imperfect Cell Action

**Published:** 1858-08

**Authors:** R. E. Haughton

**Affiliations:** Richmond, Ind.


### SELECTIONS.

*Tubercular Phthisis; the Result of Imperfect Cell Action.* By R. E. Haughton, M. D., Richmond, Ind.

In entering upon a review of the treatment which has been proposed in consumption, and before offering my own views of treatment, I remark, first, that I regard consumption as a curable disease, and for proof would refer to the pages of medicine and to the evidence of its ablest writers in the Old World, as well as the New, such as Laennec, Louis and Andral in France; to Ried, Murray, Mills, Scudamore, Coregan, Flood, Forbes, Hastings, Watson, Clark, Carswell, in England; in our own country, Parrish, Physic, Morton, Gerhard, Swett, and others, who have testified to cures. Yet, there is in the public mind, a deeply rooted conviction that it is incurable, and this conviction is strengthened by physicians, who are in the habit of saying there is no cure for consumption. But while this course is pursued, we advance none in the right direction, and sit down satisfied with the belief that this disease, more than all others, is beyond human control. Opposed to this view, let us briefly notice the opinions of some of the names just mentioned. Laennec who adopts the idea of the curability of consumption, after examining the lungs of many persons who had died of other diseases, says: "after I was convinced of the possibility of cure, in cases of ulceration of the lungs, I examined these remains more closely, and came to the conclusion that in every case, they might be considered cicatrices." "This fact, says he, seems to me to leave no doubt of the nature of these productions, and of the possibility of healing ulcers in the lungs." Says Dr. Forbes, Fellow of the Royal Society of England, and one of the principal editors of British and Foreign Medical Review, "For as many as eight or ten examples, of cicitrization of the lungs after tubercles, I refer the reader to M. Andral's Clinical Medicine, Book III, page 382. Dr. Stokes, of the Meath Hospital, Ireland, says: "we may consider the treatment of consumption under to heads, curative and palliative." There can be no doubt that as medicine advances, the cures of consumption will be much more frequent—its nature will be better understood. Dr. Carswell. the eminent professor of pathological Anatomy in the London University, says: "the cure of a disease is indicated first, by the cessation of those symptoms, which are peculiar to it, or the restoration of those modifications of function to which its existence gives rise. There must be few practical pathologists, he says, who will not consider the anatomical facts as evidence that tubercular consumption is a curable disease. And we can not, says he, avoid repeating the fact, that pathological anatomy has perhaps never afforded such conclusive evidence in proof of the curability of a disease as it has of tubercular consumption."

Dr. Gerhard, in his work on diseases of the chest, affirms, not only the curability of consumption, but the various appearances which are presented in the lungs when recovery has taken place. He says we have direct proofs of the curability of consumption. That evidence is derived from pathological investigation, and of this there is no more striking proof or illustration, than the case of an eminent physician of this city, the late Dr. Parrish. It is well known that he regarded himself as laboring under pulmonary consumption, at an early period of life. He finally recovered vigorous health, lived to the age sixty, and died of disease of the kidneys. Dr. Gerhard says that consumption is strictly a curable disease. Dr. Swett one of the physicians to the New Nork Hospital, in his treaties on diseases of the chest, affirms the curability of consumption. We look over this array of authority in favor of the curability of consumption, and yet, in vain do we find that the means resorted to prove successful. But here is the reason for the opinion that this scourge of the human race is curable. If consumption be curable by any process or effort of nature, even in unfavorable cases, then *it is* curable. And the members of the medical profession should learn, if they have not *already*, that *nature* is our teacher and guide, and it is the province of the true physician to minister at her altar, and to follow where she may lead. Then if nature can effect a cure of consumption when cavities have existed, there is a cure, and we should study the changes effected, the influences which may have wrought the sechanges, and profit thereby. I am willing to admit progress in the right direction, but we have not yet became sufficienty untrammelled from the shackles of authority and the teachings of schools, to read nature, aud investigate her remedies. The universe of God has many remedial agents which have not been tried, and even remedies which have been used and are now covered over by the oblivion of the past, gone into disuse; among those are some of the best remedial agents of the Pharmacopœia, in many diseases. Let such be brought to light, let research and experiment test the value of our agents, not experiments in diseased action upon the human body, but let our knowledge be so perfected that human life is not the sacrifice of our efforts.

Believing that consumption is a curable disease, and that the causeses, as brought to light in these papers, are capable of being removed, we come to speak of its treatment and some of the remedial agents which have been proposed. There are three great channels through which we apply our remedies. First, the stomach, the great highway to death in this disease; the skin; and lastly through the lungs themselves. The functions of the stomach are much abused, already much weakened by the direct influence of depraved blood, yet nauseous drugs are poured into it, deranging still more its functions, till at last its operations are suspended, the wheels of life are clogged and decay goes rapidly on. In more ancient days, blood-letting was resorted to as a curative agent or means; again emetics were thought to be useful, and the various alteratives and tonics have each been recommended and each gone into disrepute. It would seem as though it was all guess work, and all doubt, as to the condition of the general *health*, and of the *lungs* in *particular*, if we but look at the *treatment* for a history. There is an impairment of the general health that remedies can not remove; why then, oppress the stomach with nauseous remedies when it is highly important that the digestive power should be sustained. Exercise in the open air with the free sunlight of heaven, and the inhalation of the oxygen of the air are natures own remedies and their influence should not be prevented by close rooms and want of good ventilation. The blood is depraved, deficient in healthy elements, and these means, to a certain extent, have the power of changing the condition of the blood, and this is an important step in the treatment. Again, the stomach should digest, and the absorbents take up the elements of nutrition, and assimilation be so perfect that the additions to the blood should be more perfect in normal elements than before; and thus is accomplished, by a simple and natural process, more than all the remedial agents in the universe have as yet accomplished.— The treatment which will prove successful, must be based upon the condition of the blood, knowing the deficient elements, and knowing them to restore them to normal proportions, thus making the blood mass as nearly normal as a deficient respiration will permit. But as this process is effected, and expansion of the lungs takes place by inhalation of the good air, the blood comes to be perfected, and then we have but the local trouble to be disposed of. The influence of blood, which in healthy elements, will arrest the morbid process in the lungs and thus become curative in its circulation. Prof. Gross says, that a poor and impoverished state of the blood is most favorable to the development of tubercle. Andral, Simon and others have analyzed the blood in health and in disease In health they give us—

| | | |
|---|---|---|
| Water | 790 | grains. |
| Discs | 127 | " |
| Albumen | 80 | " |
| Fibrin | 3 | " |
| | 1,000 | " |

In anemia they give us blood changed in elements, so that in 1000 grains we have—

| | | |
|---|---|---|
| Water | 840 | grains. |
| Discs | 71 | " |
| Albumen | 80 | " |
| Fibrin | 9 | ". |
| | 1,000 | " |

We have also as the analysis of tubercle, which is a deposit from the blood, by Dr Hecht of Strasburgh—

| | | |
|---|---|---|
| Albumen | 26 | grains. |
| Gelatine | 22 | " |
| Fibrin | 34 | " |
| Water | 30 | " |
| | 112 | " |

While Thenard found tubercle to consist of 98 parts in 100 of albumen. Taking it for granted that tubercles are composed of albumen principally, and that the red corpuscles are deficient, we infer a larger amount of water than is normal in healthy blood. If this be true, we, have the peculiar dyscracia of blood belonging to tuberculosis. But we have the analysis of the blood by Simon, where the water is increased, and the other elements changed from their normal proportions—

| | | |
|---|---|---|
| Water | 807 | grains. |
| Solid Res | 192 | " |
| Fibrin | 4 | " |
| Fat | 2 | " |
| Albumen | 98 | " |
| Globulin | 71 | " |
| Hœmatin | 3 | " |

This may be taken as an example from which there is no material deviation, except so far as the increased amount of diseases adds to the changes in the blood. This analysis is made of blood, taken in the first stage of consumption. As the disease advances to its different stages, the blood becomes more and impoverished, and this is readily understood, because digestion, absorption, assimilation are all arrested or so imperfectly performed that the blood is not benefitted by any of these means.—The question comes up, how shall we improve the blood, remove the general impairment of the fluids and solids, and thus place the system in a condition to return to health and strength? We have been aiming to accomplish this in part by the use of codliver oil, but we fail because the assimilative processes are defective, and the oil does not enter the system as a curative agent in many cases, because it fails to be appropriated.—The oil does not require digestion in the sense of other injesta, but by not tasking the stomach might be converted into fat by the assimilation, and deposited throughout the body. It is easily converted into fat, and while the deposition of fatty materials are going on in the body, we have no fears as to the result. But this is not often the case, the oil proving nauseous to the stomach and thereby preventing the beneficial influence to be desired. It must be admitted that cases have been benefitted, and some apparently cured, but we experience so many failures that we look about us for some more efficient means. My own experience teaches me that those cases which have been benefitted are the accidental cases, in which there was no original failures of the powers of nutrition. It must be admitted that where the stomach has failed entirely to digest, or change even fluid injesta, as I have often seen, it would not be likely to retain or act upon the oil in such a way as to be beneficial. In one instance the stomach failed to digest, the food taken passed by the bowels as eaten, and the patient, though not far advanced in the progress of the disease in the lungs, rapidly failed, and died from actual failure of nutrition, whereas, if he could have been sustained by any means of nourishment he must have lived much longer. In such a case the oil could not have been used with benefit, and we are left to look upon the patient passing away without a remedy to stay the progress of the disease. The oil is intensely nauseous to some persons, and sickness of the stomach is prostrating. It impairs the nervous power, already weakened and failing, hence it is useless and contra-indicated. Again, it is well known that there is deficient or arrested biliary secretion in this disease, hence the oil can not be absorbed so well, as a proper admixture of bile with any oleaginous element of food, fits it for the action of the absorbents.—There is no digestion of oils or fats, so to speak, and intimate admixture of all the fluids of the digestive organs, which favors a minute division of the oily particles, favorable for lacteal absorption. From observation, this, very often, does not take place in those cases where failure of nutrition is the first and most prominent lesion, hence the oil fails of its desired effect. In consumption, the principal agents or elements of food are to be drawn from the saccharine and oleaginous group, because either are convertible into fat, and those of the saccharine group are useful in the process of combustion and the generation of animal heat, which is an important item in the consumptive person, who is always chilly, except in heated rooms, the very place of all others, perhaps, in which he ought not to be found, and which are decidedly prejudicial to his present and future progress towards health. There are other modes of treatment which have the prestige of great names, and which are held up to the people and the profession as *the* modes which are to be successful. But alas, they can hardly show a case of tubercular consumption (purely so) cured. The injection of nitrate of silver into the cavities of the lungs and the cure of bronchitis and laryngitis by the same means. Laryngitis may be cured, but the passage of a sponge probang below the rima-glottidis, is in my opinion an impossibillity, and even were it possible, highly dangerous and hazardous to the patient, and the effort to effect it evincing great temerity upon the part of the physician. Again, it is possible that an expert operator may introduce a curved instrument, in the shape of a catheter, through the larynx, pass the rima of the glottis, and thus enter the trachea, but after reaching the trachea, the instrument must pass some distance to the division of the bronchia, and then to reach a cavity, must diverge and pass still farther. This requires time, and is a dangerous experiment for one suffering with cavities in the lungs, as has been proved in the death of some of the subjects of this operation.—Again, the lungs were not made to be thus invaded by foreign bodies, air being the proper and only thing intended to enter them and reach the most minute divisions of the bronchial tubes, and each minute air-cell which they contain. There is a proper adaptation and combination of the elements of the air to meet all the conditions and necessities of the animal economy, and the lungs are so constructed and arranged that air is the only thing which does not give rise to irritation when received into the lungs, hence; the introduction of a foreign body, or an effort to do so, below the vocal chords, with the view of removing diseased conditions of the structure of the lungs, I regard as opposed to sound pathology, and in direct opposition to the natural law of respiration, the function of the lungs as dependent upon air, and the resistance expressed when a foreign body has accidentally been found in the air-passages. Also, that there is no satisfactory evidence that cavities have ever been injected, and of course, none of their cure by this process. Hence, I regard this plan of treatment as futile in the treatment of tubercular consumption with cavities. There is too much, and has been, heretofore, tco much of a blind adherence to the theories and doctrines of men in high places, men who should be the exponents of truth in their teachings, rather than mounting some hobby to mislead the younger and more timid of the profession. Humanity demands at our hands the advancement of truth and the eradication of error, and we should not hold him guiltless who by virtue of position, should teach error instead of truth. In speaking of the modes of treatment, I refer to some which now occupy some considerable space in the public mind, and I might say that some degree of confidence has been reposed in them. The doctrine of inhalation is one of those, and though at first my mind was somewhat favorably impressed with the specious plausibility of the teachings of one who is regarded as the exponent of this doctrine, yet after more than two years careful investigation of the subject, I regard it as an empty bubble, which will glitter for a while upon the waves of time and then burst, leaving no trace of its existence, save the vacant place, once occupied by those who have gone to their eternal homes, the victims of disease and hope deferred, under this treatment. The doctrine of inhalation is not new, it was proposed in the time of Hippocrates, 400 years before the Christian Era. Yet, it has been taught as a new fact, a brilliant discovery of these later days, by men who excel in wisdom above that which is written. The cause of tubercle is said to be, by the exponents of inhalation, "a deficient supply of air to the lungs." It is admitted upon all hands that this is an important influence, but we regard it as an error to say that this is the only cause. If this were true, all that would be necessary. would be to supply the proper amount of air, of a proper quality, to effect a cure in most cases. Not so. The fact that consumption grows out of indigestion frequently, when the lungs have never been impaired, shows conclusively that a morbid assimilation of food is a cause of consumption. An objector may say that there was imperfect respiration during the time. Not so. The broad chested, herculean frame of the blacksmith, the farmer, are the victims of this form of disease, while exercising in the open air and at the anvil. They have impaired digestion and assimilation, and the blood contains the elements of tubercle, which the respiration of healthy lungs can not burn off or remove. There are hereditary influences, also, at work, which are productive of this disease. Impure air, morbid assimilation, take their rank as alternate causes, sometimes the one prevailing first as a cause, and sometimes the other. Also, hereditary transmission, and a host of other influences might be mentioned as causes of this disease. But the most prominent have been mentioned, and now from the causes we deduce a modus operandi in the cure. Just as we determine a cause to be most prominent, just so we regulate the mode of treatment. In proportion as a cause is active, just so must the counteracting forces be active. The basis of treatment in inhalation, is that the remedies are applied to the diseased surface. But we are told that this disease is both a constitutional and a local disease. The constitutional disease is one of the blood-mass, and the local disease, the result of this disease of the blood. Then the first indication is to purify the blood and admit pure air. But inhalation says it purifies the blood and removes the local disease at one and the same time. But it proves too much. The want of pure air is, according to inhalation, *the* cause. But now, instead of introducing air pure and unadulterated, it is mixed with various medicinal vapors, inhaled, and thus, forsooth, the blood-mass is to be purified. Alas, for human wisdom. Air is the only agent which purifies the blood, when introduced into the lungs—all else besides deteriorates and impairs. Then we come to this conclusion, that there is an error in the doctrine, and for proof let us look at its results as at present it is applied. There is no institution in the country, hospital, or anything which deserves the name, in which this treatment is solely tried and tested, and in the absence of such institutions, we have to rely upon individual experience without any arranged statistical facts. We are told in the journals of the day it is eminently successful. What are the facts? Men for pecuniary gain have become their own editors, and puff their own cases, and themselves in particular, giving names and cases which have no existence in fact, and which are said to have been cured by this plan, thus inducing many poor sufferers to risk their all under the dulusive hope of being cured. The remedies are classified under five heads; expectorant, anodyne, astringents, antispasmodic, alterative, &c. These remedies are calculated to act as palliatives, quieting the patient, soothing irritation, cough, and thus the patient is still hoping, with hope deferred; and there are no cures well attested as tubercular consumption, with cavities in the lungs, where the cure can be confidently attributed to the use of these remedies.—"Alterative inhalations are said to be of chief importance in the treatment of consumption, 'properly administered and combined, these several remedies constitute a treatment which strikes at the root of the monstrous error which for centuries has held the reason of mankind in blind submission to custom and authority.'" This is the language of inhalation, by exponents. I would for the sake of humanity it were so. To sum, up then, in a few words—it is palliating—it is not curative; because it begins wrong, and violates the first essential doctrine of its theory. It does not purify the blood, it does not furnish pure air to the lungs, and in these conditions are bound up the results of treatment. It then should, so far as truth is concerned, be submitted to trial in hospital practice, and the facts should be arranged as statistics, and if truth is in it, let the world receive it, and if errror reject it.

But now I propose briefly, a few remarks in reference to the treatment of consumption, as deduced from the pathological conditions which are adv..nced in the previous papers upon this subject.* I regard consumption as both a constitutional and local disease, the local disease being the result of the constitutional Then the mode of cure is to arrest the general disease by removing the causes. Impairment of the blood is the chief condition which gives rise to manifestation of symptoms and the means which restore it to a normal condition, and the system to such a condition as shall manufacture good blood, are the curative means. Fresh air, exercise, and good digestion and assimilation are among the important means.—Drugging the stomach will never do this—an organ already frequently impaired by excesses of various kinds. Then why derange it still more. There may be times when a tonic will do much good if wisely selected, but the indiscriminate medication in this disease is an evil to be greatly deplored. In impairment of the stomach, pepsin may be introduced with the view of assisting digestion, as this article possesses the power in combination with the acids of the gastric juice, of dissolving articles at a proper temperature out of the stomach. The experiments of Wasman † upon this subject, are to the point. Next, the introduction of such food as can be easily digested and assimilated, and which contains the elements of repair in their best combination to suit the condition of the functions of absorption and assimilation. By this means we change the blood mass, and oxygenation is perfecting the great work of the pefect assimilation of the chyle into blood. Bnt again, if we fail because of the structural change in the lungs, or because digestion and assimilation are not performed, we can do more. I now propose *transfusion of blood* as affording a means of changing the blood-mass, thereby changing the conditions of the system, reïnvigorating the brain, and sending out increased nervous energy, and promoting digestion and assimilation and thereby placing the system in a condition to return to health and vigor. If we thus change the blood mass, tubercle ceases to be deposited. When it ceases to be deposited, the great work of cure is partly accomplished, and then the removal of those already deposited, or rendering them in-

* (Nashville Journal.) (Medical Independent.)
† Carpenters Physiology.

active and latent, or producing absorption, completes the cure. If cavities have been formed, by supporting the general health in this way, these cavities will soon cicatrize, and a cure is the result. It is well known that tubercular depositions take place by successive crops, and softening in the same way, hence, arrest the deposit by changing the blood, and if softening of the first crop takes place, that is the end of it, no more to soften. And how many pass through several periods of softening, with the increasing irritation still added till worn out, by the continual destructive change, which thus takes place in the lungs. I do not know that transfusion of the blood in consumption was ever thought of or recommended in the treatment of this disease, but I arrive at it from the pathology, which I regard as true, and it recommeds itself because it is the *thing* the diseased system can not do for itself, change its own blood, or manufacture it anew, because of the failure of the blood manufacturing powers of the economy. Iron is one of the elements of healthy blood, and this is deficient in all those cases, and which in transfusion is directly furnished in its proper relations, and can be thus appropriated to its proper use in the economy. So it is with the other elements thus furnished, and we have the very thing at once effected, which has so long failed to be done in any other way. Transfusion is successful in cases of hemorrhage, where the system is exhausted and anæmiated, and why not be successful in other cases where the blood is diseased, and the cause of the *local disease.* Some would object to this, because the patient should not be subjected to such a process. The irritation is small compared with the irritation often kept up with drugs, from day to day, without any corresponding benefit. This plan or mode of treatment must give strength because from the blood are deposited all the tissues, and all the secretions are from the blood, and if this be a pure article, then must all the results flowing from it be good, "for it is written, 'the blood thereof is the life thereof.'" Again, show me a patient whose system manufactures good blood, even if the lungs are diseased, and I will show you one who is not very likely to die of consumption. When the function of the lungs are interfered with, I know very well that the blood is not well aërated, and there is a tendency to deterioration of the blood, and if there is no effort at expansion, the deterioration will go on. For the mode of performing transfusion, the plan proposed by Ramsbotham, in his work on the Process of Parturition, page 333, is sufficient. The history of transfusion dates back to more ancient days, and afterwards fell into disuse, till the practice was again restored by Dr. Blundell,* in cases

*(Physiological and Pathological Research, 1824.)

of dangerous and copious hemorrhage, in lying in women. The names of Hamilton, Davis, Velpeau, are referred to in reference to this subject; as also, Denman, Leacock, and Lane. That transfusion is not circumscribed in its benefits to one form of trouble is quite certain, and that it has never been before tried or suggested in any case of scrofula or consumption that I am aware of, and it has quite as much to recommend it in those cases, as in those where it has been used, I am quit certain. The experiments upon which the doctrine of transfusion is founded, were performed by Dr. Leacock, and made known in an inaugural thesis, published in Edinburg in 1817, and afterwards the experiments were repeated and varied by Blundell. The experiments prove that healthy human blood is alone fit for the purposes of transfusion, and this is of, course, eminently true in the condition of the blood which obtains in consumption. But leaving *this* for the present, we have a few suggestions to make in reference to some of the other means which may be used beneficially in the treatment of cases of consumption. In place of codliver oil, I find that rich cream or very rich milk answers a much better purpose, from the fact that it does not nauseate the stomach, and contains the oil or fatty elements which are easily and beneficially appropriated to the uses of the system, without impairing digestion and assimilation. As a tonic, some preparation containing iron is evidently best suited to most cases of tubercular disease, and frequently improves the appetite and digestive power. I am treating a case at the present time, in which the Syrup Iod. Ferri. is most strikingly beneficial, a few does of which, in this case, improves the appetite when almost entirely deficient, and beef will be craved and apparently well digested. This preparation of iron, in another case of a child, where both scrofula, as manifested in glandular enlargements, and also a diseased condition of the lungs existed, acted like a charm in removing the morbid conditions present. Exercise in the open air every day, and rich diet, as cream, butter and the gravy of beef and pork was allowed ad libitum, and at the present time the child is fat and hearty, cough gone, emaciation gone, glandular enlargements gone, and the child bids fair for long life, as any child of its age. Of the various complications of consumption I have not spoken, and of some of the forms of disease which often precede it and lead to its development, when not arrested by treatment. These subjects are not spoken of in the previous papers of which this is a part and the conclusion. Of these I may treat hereafter, and of the modes of the treatment which may successfully counteract and cure after development of the disease. I shall not continue at present, any views upon this subject, but ask for that part of treatment by transfusion in tubercular diseases, a candid investigation of medical men, and for the pathology upon which it is based, refer them to the November and January Numbers of the Medical Independent, Detroit. There are corresponding evidences of the value of a similar treatment by the use of the blood of animals in the treatment of tubercular hemorrhage, of the same in the exhaustive diseases of children, where it has been given as an internal remedial agent, subject to the process of assimilation. The authority in these cases are sufficient, and the evidence of benefit and cure well authenticated by the medical journals of foreign publications.—*Cincinnati Lancet & Observer.*

